# Enhancement of Mycophenolate Mofetil Permeation for Topical Use by Eucalyptol and N-Methyl-2-pyrrolidone

**DOI:** 10.1155/2016/9672718

**Published:** 2016-03-16

**Authors:** Thanaporn Amnuaikit, Chalermkiat Songkram, Sirirat Pinsuwan

**Affiliations:** ^1^Department of Pharmaceutical Technology, Faculty of Pharmaceutical Sciences, Prince of Songkla University, Hat Yai, Songkhla 90112, Thailand; ^2^Department of Pharmaceutical Chemistry, Faculty of Pharmaceutical Sciences, Prince of Songkla University, Hat Yai, Songkhla 90112, Thailand

## Abstract

Mycophenolate mofetil (MMF) is a prodrug of mycophenolic acid (MPA) which can be metabolized by esterase. MMF has been approved by the United States Food and Drug Administration (USFDA) for treatment of psoriasis patient with skin symptoms. However, it remains unclear whether MMF is efficiently effective to treat skin symptoms developed from psoriasis. The insufficient amount of MMF penetrating through the skin results in the treatment failure due to the difficulty in MMF penetration through the stratum corneum. Skin permeation enhancers such as eucalyptol (EUL) and N-methyl-2-pyrrolidone (NMP) potentially aid in increasing skin penetration. This study aimed to investigate the effects of a concentration ratio (% w/v) between two enhancers (EUL and NMP). The results showed that EUL enhanced MMF permeation with an enhancement ratio (ER) of 3.44 while NMP was not able to promote the penetration of MMF. Interestingly, the synergistic effect of the two enhancers was observed with a suitable ratio given that the ER was 8.21. EUL and NMP are promising enhancers for the development of MMF based skin product.

## 1. Introduction

**Figure 1:**
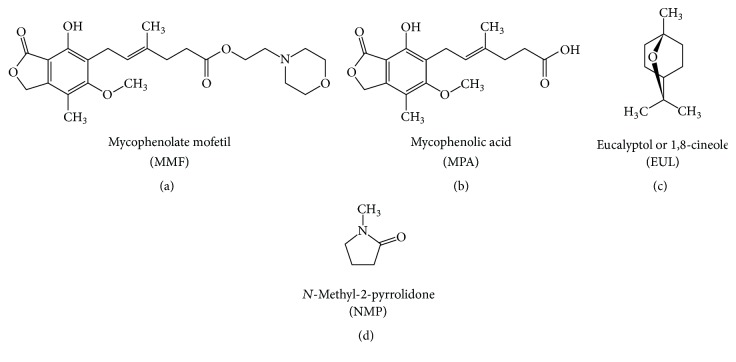
Chemical structure of MMF (a), MPA (b), EUL (c), and NMP (d).

Mycophenolate mofetil (MMF) is a morpholinoethyl ester derivative of mycophenolic acid (MPA) (Figures 1(a) and 1(b)). It is metabolized by esterase resulting in the MPA as an active compound. Many clinical studies showed that oral MPA therapy in the treatment of moderate to severe psoriasis has been both effective and safe since the early 1970s [1]. However, MPA still has oral bioavailability problem. Therefore, MMF was developed as a prodrug in order to improve MPA bioavailability. The USFDA has approved MMF as a psoriasis drug since 1995. MMF is commonly marketed under the trade name Cellcept® (Roche for oral use).

MMF is an immunosuppressive drug used in the management of autoimmune disorder [2, 3] such as systemic lupus erythematosus (SLE) [4], scleroderma, and immune-mediated skin diseases. MMF is chosen to treat psoriasis as well as other autoimmune diseases. Psoriasis is caused by the malfunction of T lymphocytes which stimulate keratinocyte cell division resulting in the rapid growth of skin cells and the abnormality of an epidermal layer of the skin. Psoriasis leads to skin inflammation and exfoliation with pain [5].

Nowadays, the skin products for treatment of psoriasis in patients including steroid and vitamin D3 cream still do not control the skin symptom well. Several studies have reported the application of MMF and MPA for skin treatment. Shoji and colleague [6] developed MPA topical formulation for treatment of allergic contact dermatitis induced by dinitrofluorobenzene. Their results showed the effective treatment. Moreover, they suggested that MPA topical formulation can potentially be used to treat psoriasis. Wohlrab et al. [7] revealed that 2% (w/w) of MMF cream applying on the skin could control skin symptoms very well. In contrast, Geilen and Mrowietz [8] reported that MPA ointment had no effect on psoriasis. This was due to the insufficient amount of the drug in the skin. Although there were several reasons for the failures in psoriasis treatment, the insufficient drug permeated through the skin is challenging in the development of MMF and/or MPA skin product with enhancing skin permeation.

Skin enhancer is one of several solutions to overcome the permeability problem of MMF. However, there are a lot of enhancers which are classified based on their chemical structures. Songkro [9] categorized skin enhancers into 17 groups according to their functional groups and chemical structures including pyrrolidones, glycols, and terpenes. Each group of enhancers displays its own mechanism of action in promoting skin permeation. Enhancers commonly used in skin product because of the high effectiveness and low toxicity are eucalyptol, N-methyl-2-pyrrolidone, propylene glycol, nonionic surfactant, and so forth [10].

In this study, we aimed to determine the skin enhancing ability of the selected enhancers which are eucalyptol (EUL; Figure 1(c)) and N-methyl-2-pyrrolidone (NMP; Figure 1(d)) representing terpenes and pyrrolidones groups, respectively. We hypothesized that the combination of two or more enhancers with different mechanism of action (synergistic effect) is more effective and safe in comparison to the use of them alone [10]. We determined the enhancement ratios (ER) as well as the skin permeation flux. Furthermore, we investigated the ratio between the two enhancers used in both single and combination formulation.

## 2. Materials and Methods

### 2.1. Materials

MMF was obtained from the extraction of Cellcept capsule (Roche Thailand Ltd.). MPA was synthetized by hydrolysis of MMF. The identification of the extracted MMF and the semisynthetic MPA was confirmed by high performance liquid chromatography (HPLC), infrared spectroscopy (IR), and nuclear magnetic resonance (NMR). The secondary standard of MMF and MPA was a gift from the Molecular Pharmaceutical Research Center (Faculty of Pharmaceutical Sciences, Prince of Songkla University, Hat Yai, Songkhla, Thailand). Acetonitrile (HPLC grade), methanol (AR grade), phosphoric acid, monobasic sodium phosphate, dibasic sodium phosphate, and sodium chloride were purchased from Labscan Asia Co., Ltd. (Bangkok, Thailand). Diethyl ether n-hexane, 95% ethanol, and chloroform were of commercial grade and purchased from High Science Limited Partnership (Songkhla, Thailand). EUL (1,8 cineole), NMP, and triethylamine were purchased from Sigma-Aldrich (Denmark).

### 2.2. Preparation of MMF

The amount of 40 capsules of Cellcept was used as a starting material. One capsule contained 250 mg of MMF and other excipients such as diluent and glidant. MMF was extracted by chloroform. The insoluble matters were separated. Chloroform was evaporated out by rotary evaporator. MMF was recrystallized in diethyl ether-n-hexane to obtain a pure compound. The crystal of MMF was identified by its melting point, chemical structure using IR, ^1^H-NMR, and ^13^C-NMR spectrophotometry to confirm the purity of MMF.

### 2.3. Quantitative Analysis of MMF and MPA by HPLC

The amounts of MMF and MPA in the skin permeation study were analyzed by high performance liquid chromatography (Agilent 1100 series, Palo Alto, CA). A reverse phase, BDS HYPERSIL C18 (250 × 4.6 mm, 5 *μ*m), was used. A chromatographic system was acetonitrile: 0.1% phosphoric acid + 0.1% trimethylamine (55 : 45 v/v) at 1.0 mL/min. A UV detector was set at 220 nm. The injection volume was 10 *μ*L. This method was modified from Heard et al. [11].

The standard solutions of MMF were prepared in ethanol in a dilution: 5.0, 2.0, 1.0, 0.5, and 0.25 *μ*g/mL. Because MMF is metabolized resulting in the MPA when applying on the skin, the MPA standard concentration was also prepared similar to that of MMF. MMF and MPA standard curves were conducted and used to relatively calculate the amount of MMF and MPA in the skin permeation study.

### 2.4. *In Vitro* Skin Permeation Study

The effects of MMF and enhancers concentrations on skin permeation were measured through newborn pig skin using modified Franz diffusion cells. Moreover, the synergistic effect of enhancers' combination was determined. The full thickness flank skin was taken from naturally dead newborn pigs, weighing 1.4 to 1.8 kg. The newborn pigs were freshly provided by a local pig farm in Songkhla Province regulated by the Department of Livestock Development, Thailand. The full thickness flank skin was prepared as previously described [12]. Briefly, the epidermal hair was removed without damaging the skin and excised with a scalpel. The subcutaneous fat and underlying tissues were carefully removed from the dermal surface. The isolated skin was mounted on a modified Franz diffusion cell with the stratum corneum facing upwards. The modified Franz diffusion cell is composed of two compartments: a donor compartment containing drug solution sample or preparation and a receptor compartment containing receptor fluid which represented blood circulation. The effective diffusion area of the diffusion cell was 1.77 cm^2^. The receptor compartment was filled with 11 mL of phosphate buffer saline solution pH 7.4 (PBS). One milliliter of each drug solution sample was applied on the skin surface in the donor compartment. The Franz diffusion cells were maintained at 37°C with stirring at 200 rpm throughout the experiment. A sample (1 mL) of receiver medium was withdrawn through the sampling port of the diffusion cell at 1-, 2-, 4-, 6-, 12-, 18-, and 24-hour time intervals. An equal volume of fresh PBS was replaced into the receptor compartment after each sampling. All withdrawn samples were analyzed by HPLC technique. The cumulative amount of drug that permeated the skin was calculated by the following equation:

(1) $$\begin{array}{c} Q_{t}=V_{r}C_{t}+\overset{t-1}{\underset{i=0}{\sum }}V_{s}C_{i}, \end{array}$$

where *Q*_*t*_ was the cumulative amount of drug permeation, *C*_*t*_ was the drug concentration of the receptor fluid at each sampling time, *C*_*i*_ was the drug concentration of the *i*th sample, and *V*_*r*_ and *V*_*s*_ were the volumes of the receptor fluid and the sampling volume, respectively.

To study effect of MMF concentration on skin permeation, MMF concentrations were prepared at 36, 76, 160, and 300 *μ*g/mL by simply mixing with 3% v/v of ethanol in water. Each enhancer (EUL or NMP) was incorporated to 300 *μ*g/mL of MMF solution with various % w/v concentrations; 2.5, 5, 10, and 20 in order to determine the effect on skin permeation. Moreover, a combination of concentration ratio between EUL and NMP was prepared (% w/v), 5 : 5, 5 : 10, 10 : 5, and 10 : 10 and incorporated to 300 *μ*g/mL of MMF solution before applying to the donor compartment.

### 2.5. Statistical Analysis

The data were presented as mean ± SD (*n* = 3–6) and statistical analysis of skin permeation fluxes of MMF and MPA was performed using Student's *t*-test and ANOVA. The level of significance was taken as *p* ≤ 0.05.

## 3. Results and Discussion

### 3.1. Preparation and Identification of MMF

**Figure 2:**
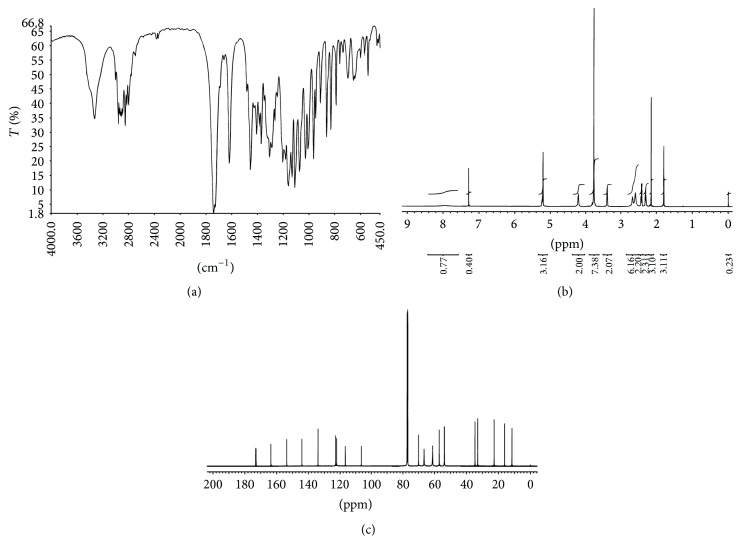
Identification of MMF by IR (a), ^1^H-NMR (b), and ^13^C-NMR spectrum (c).

MMF was extracted from Cellcept 40 capsules from other excipients by simple solution extraction technique and purified by a suitable solvent for recrystallization. The amount of pure MMF was approximately 9.5 g of total drug powder (10 g) with 95% yield. The appearance of crystal was colorless. The melting point was in the range of 91–93°C that was consistent with a standard reference of that of 93-94°C. The identification of MMF was analyzed and showed in Figure 2: IR spectrum (Figure 2(a)), ^1^H-NMR spectrum (Figure 2(b)), and ^13^C-NMR spectrum (Figure 2(c)). The extraction and purification method of MMF from the capsules yielded a quantitative amount and the quality of MMF. The informative details of IR spectrum, ^1^H-NMR spectrum, and ^13^C-NMR spectrum were shown as follows.

IR (KBr): 3329 cm^−1^ (*ν*_O-H_), 2801–2960 cm^−1^ (*ν*_C-H_), 1740 cm^−1^ (*ν*_C=O_, esters), 1725 cm^−1^ (*ν*_C=O_, esters), 1619–1456 cm^−1^ (*ν*_C=C_), 1240 cm^−1^ (*ν*_C-C-O_), 1205 cm^−1^ (*ν*_C-O-C_), 1076 cm^−1^ (*ν*_O-C-C_).

^1^H-NMR (500 MHz) C*D*Cl_3_: 1.80 (*s*, 3*H*, C*H*_3_-C=), 2.15 (*s*, 3*H*, C*H*_3_-Ar), 2.29–2.32 (*m*, 2*H*, C*H*_2_-C[CH_3_]=), 2.41–2.44 (*m*, 2*H*, C*H*_2_CO), 2.60 (*m*, 4*H*, 2C*H*_2_-N), 2.67 (*m*, 2*H*, C*H*_2_-N), 3.39 (*d*, *J* = 6.8 Hz, 2*H*, C*H*_2_-Ar), 3.77-3.77 (*m*, 4*H*, C*H*_2_O), 3.77 (*s*, 3*H*, OC*H*_3_), 4.21 (*t*, *J* = 5.0 Hz, 2*H*, C*H*_2_O), 5.21 (*s*, 2*H*, C*H*_2_O), 5.22–5.24 (*m*, 1*H*, C*H*=), 7.97 (*br*,* s*, 1*H*, ArO*H*).

^13^C-NMR (500 MHz)* C*DCl_3_: 11.5, 16.1, 22.6, 32.9, 34.6, 53.7, 56.9, 61.0, 61.3, 66.5, 69.9, 106.4, 116.6, 122.1, 122.8, 133.9, 144.1, 153.6, 163.5, 172.8, 173.1.

### 3.2. Quantitative Analysis of MMF and MPA

**Figure 3:**
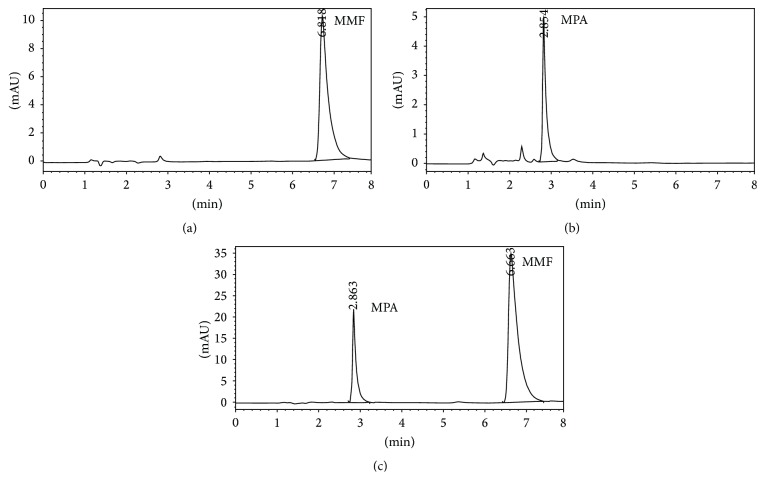
Chromatogram of MMF (a), MPA (b), MMF, and MPA in donor solution (c).

Quantitative analysis of MMF and MPA by HPLC was showed in Figures 3(a) and 3(b), respectively. The retention time of MMF was 6.755 ± 0.040 while MPA was that of 2.860 ± 0.026 minutes corresponding to the reference standards. In the* in vitro* skin permeation study at 24 hours, the drug solution on the skin which remained in donor compartment was taken to analyze. The chromatogram of its solution showed peaks of MMF together with MPA (Figure 3(c)) implying that certain amount of MMF is metabolized to MPA when applying on the skin. The linearity of calibration curves of MMF and MPA was determined by plotting the peak area ratio of MMF and MPA over their concentration range of 0.25–5 *μ*g/mL indicating the correlation coefficients (*R* ^2^) at 0.9896 and 0.9991, respectively.

### 3.3. Determination of* In Vitro *Skin Permeation Values

**Figure 4:**
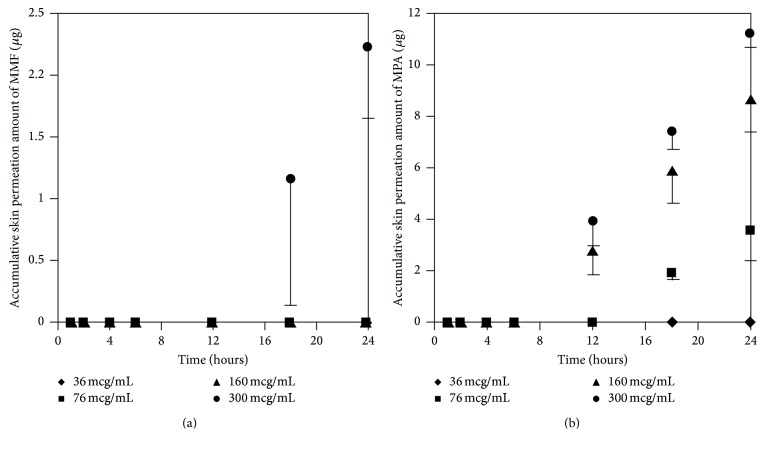
Skin permeation profiles of MMF (a) and MPA (b) at various concentrations of MMF preparation (36, 76, 160, and 300 *μ*g/mL).

**Figure 5:**
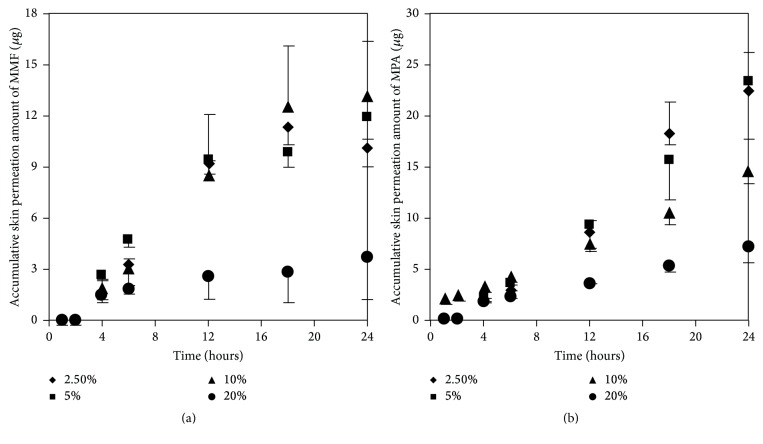
Skin permeation profiles of MMF (a) and MPA (b) at various concentrations of EUL (2.5, 5, 10, and 20 w/v) incorporated to 300 *μ*g/mL of MMF preparation.

**Figure 6:**
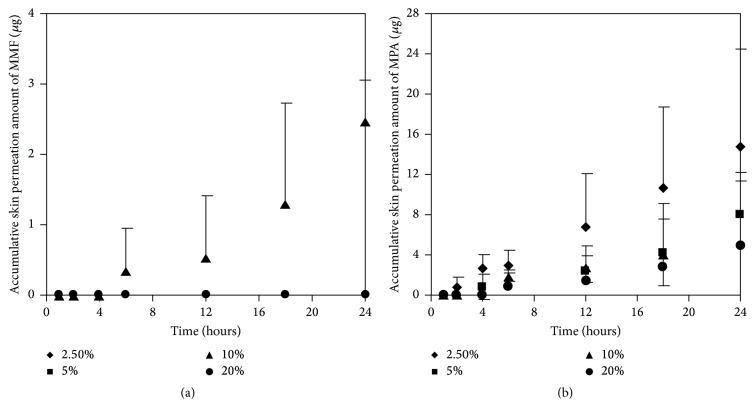
Skin permeation profiles of MMF (a) and MPA (b) at various concentrations of NMP (2.5, 5, 10, and 20 w/v) incorporated to 300 *μ*g/mL of MMF preparation.

**Figure 7:**
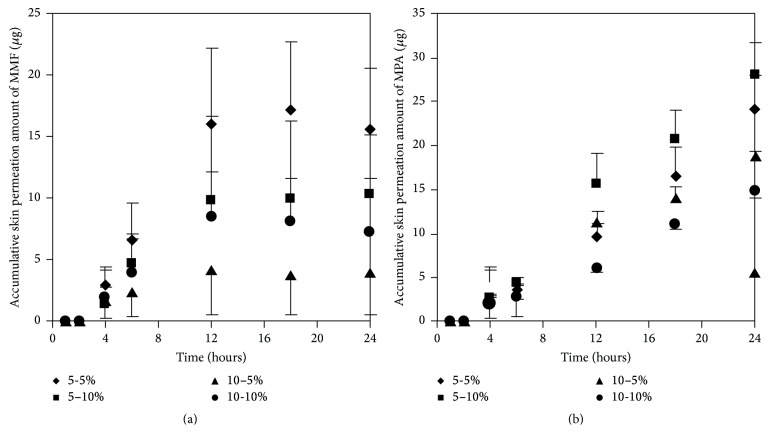
Skin permeation profiles of MMF (a) and MPA (b) at various concentration ratios of mixed enhancers between EUL and NMP incorporated to 300 *μ*g/mL of MMF preparation.

In this study, various parameters affected skin permeation of MMF including concentrations of MMF as well as concentrations and ratios of enhancers. Determination of skin permeation values in terms of skin permeation flux, ER, and lag time was used to evaluate the effect of drug concentrations, enhancers concentrations, and combination ratios of enhancers. The value of skin permeation flux (*J*) in the steady state region was obtained from the slope of the linear plot of the accumulative drug amount permeated per unit time. The lag time was obtained by experimental calculation of the *x*-intercept of the best fit line of the steady state data points of skin permeation profiles. Therefore, the values of *J* and lag times were defined from skin permeation profiles of Figures 4–7. The enhancement ratio (ER) was calculated from the ratio of MPA or MMF flux in the presence and absence of enhancers:

(2) $$\begin{array}{c} \text{ER}=\frac{\text{Flux-MPA or MMF with-enhancer}}{\text{Flux-MPA or MMF with-noenhancer}}. \end{array}$$

#### 3.3.1. Effect of MMF Concentrations on Skin Permeation of MMF and MPA

At the concentration of 300 *μ*g/mL of MMF, both MMF and MPA were found in the receptor fluid. In contrast, at 36 *μ*g/mL of MMF, neither MMF or MPA was found in the receptor fluid. Skin permeation profiles of MMF and MPA in the receptor fluid were shown in Figures 4(a) and 4(b), respectively. Skin permeation flux and lag time of MMF and MPA were shown in Table 1. The results showed that more than 76 *μ*g/mL of MMF was able to pass through the skin and all of them were metabolized to MPA due to the esterase located in the skin [13, 14] hydrolyzed MMF to MPA. However, at 300 *μ*g/mL of MMF, the amount of esterase was not enough to metabolize all of MMF completely [13]. Thus, MMF was found localized with MPA in receptor fluid when sampling the fluid out for quantification. In addition, the power of concentration gradient (160 and 300 *μ*g/mL) affected the lag time which could decrease the lag time of drug permeation. Hence, the concentration at 300 *μ*g/mL of MMF preparation was selected for applying on the skin in the donor compartment to further study the effect of enhancers.

#### 3.3.2. Effect of Enhancers Concentrations on Skin Permeation of MMF and MPA

As mentioned earlier, at 300 *μ*g/mL of MMF preparation, we found both MMF and MPA in the receptor fluid. The results showed that EUL at 2.5 and 5% w/v increased skin permeation of MMF and MPA higher than that at 10 and 20% w/v (Figures 5(a) and 5(b)). The skin permeation flux and ER values of both drugs indicated the similarity in the enhancing ability of EUL (Table 2). At 10% w/v, EUL promoted the penetration of MMF at the highest skin flux. However, it decreased skin permeation of MPA. The decrease in skin permeation of both drugs was affected by EUL at the highest concentration of 20% w/v. In the case of NMP (Figures 6(a) and 6(b)), it was unable to promote skin permeation of MMF and MPA. In addition, increasing NMP concentration tended to decrease the ability of MPA to permeate through the skin. MMF was found in the receptor fluid when 10% w/v of NMP was present. The enhancing effect of both enhancers on the skin permeation was due to a decrease in lag time implying that both drug substances could pass through skin faster than when no enhancer was present (Table 2). These results indicated that enhancers provide the advantages for improving MMF/MPA based skin product.

#### 3.3.3. Effect of Concentration Ratios of Two Enhancers on Skin Permeation of MMF and MPA

We selected two enhancers from different group of chemical structures. These two enhancers have different mechanism on enhancement of skin permeation. The synergistic effects of the two enhancers potentially occur and extensively promote skin permeation of the drug with low toxicity and low irritation [10, 15]. We observed that most of the concentration ratios of mixed enhancers increased skin permeation and decreased the lag time of both MMF and MPA (Figure 7 and Table 3). The highest skin permeation flux and ER values of MMF were affected by a concentration ratio of 5 : 5 (% w/v) while the highest skin permeation flux and ER values of MPA were affected by 5 : 10 (% w/v) of mixed enhancers. In this study, MMF might be metabolized to MPA by esterase located at the skin when applying on the skin [13, 14] resulting in the unpredictable amount of MPA depending on the amount of esterase in the skin. Although MMF is selected as a drug candidate to overcome the penetration difficulty because of its lipophilic structure, skin permeation of MMF is still at low level. Therefore, many approaches have been used to circumvent the penetration issue. Applying the synergistic effect of the two enhancers suggests a cost-effective strategy in developing MMF/MPA based skin product. EUL or 1,8-cineole is an enhancer displaying a terpene structure which acts on the interstitial lipid of stratum corneum to enhancer drug permeation whereas NMP has good solubilizing property suitable for promoting both hydrophilic and lipophilic drug to be dissolved in the stratum corneum [10]. Both of them display synergistic effect for both MMF and MPA as observed from enhancing skin permeation.

## 4. Conclusions

The skin permeation of MMF is affected by several factors. The first factor is the concentration gradient of the drug; the higher the concentration, the higher the permeation. Next, selection of a suitable skin enhancer can increase the drug permeation through the skin. Furthermore, when using more than one enhancer, a suitable concentration ratio between the two different enhancers results in a synergistic effect as observed in this study when EUL and NMP were used. The effect of skin enhancer to promote the permeation is a promising platform for future study in order to develop the skin products for treatment of skin-related diseases. However, the concentration ratio of the mixed enhancers is considered the most effective factor while other factors including clinical efficacy and skin irritation evaluation should be taken into account to further improve the formulation.

## Figures and Tables

**Table 1:** The skin permeation flux and lag time of MMF and MPA at various concentrations of MMF preparations.

MMF concentration (*μ*g/mL)		Flux (*μ*g/h/cm^2^)	Lag time (h)
36	MMF	0	0
	MPA	0	0
76	MMF	0	0
	MPA	0.37 ± 0.10^*∗*^	12
160	MMF	0	0
	MPA	0.61 ± 0.07	6
300	MMF	0.23 ± 0.05	12
	MPA	0.78 ± 0.03	6

Results are expressed as the mean ± SD of at least three experiments. There was a significant different value of skin permeation flux of MPA, ^*∗*^ *p* ≤ 0.05 when compared among various concentrations of MMF solution (76, 160, and 300 *μ*g/mL).

**Table 2:** The skin permeation flux, ER, and lag time of MMF and MPA at various concentrations of eucalyptol and N-methyl-2-pyrrolidone.

	No enhancer		Eucalyptol (% w/v)											
	MMF 300 *μ*g/mL		2.5			5			10			20		
	Flux (*μ*g/h/cm^2^)	Lag time (h)	Flux (*μ*g/h/cm^2^)	ER	Lag time (h)	Flux (*μ*g/h/cm^2^)	ER	Lag time (h)	Flux (*μ*g/h/cm^2^)	ER	Lag time (h)	Flux (*μ*g/h/cm^2^)	ER	Lag time (h)
MMF	0.23 ± 0.05	12	0.65 ± 0.06^*∗∗*^	2.79	2	0.65 ± 0.05^*∗∗*^	2.75	2	0.81 ± 0.18^*∗∗*^	3.44	2	0.17 ± 0.12	0.74	2
MPA	0.78 ± 0.03	6	1.36 ± 0.18^*∗*^	1.74	2	1.32 ± 0.28^*∗*^	1.69	2	0.69 ± 0.04	0.88	2	0.37 ± 0.06^*∗*^	0.47	2

	No enhancer		N-Methyl-2-pyrrolidone (% w/v)											
	MMF 300 *μ*g/mL		2.5			5			10			20		
	Flux (*μ*g/h/cm^2^)	Lag time (h)	Flux (*μ*g/h/cm^2^)	ER	Lag time (h)	Flux (*μ*g/h/cm^2^)	ER	Lag time (h)	Flux (*μ*g/h/cm^2^)	ER	Lag time (h)	Flux (*μ*g/h/cm^2^)	ER	Lag time (h)

MMF	0.23 ± 0.05	12	0	0	0	0	0	0	0.14 ± 0.03	0.62	6	0	0	0
MPA	0.78 ± 0.03	6	0.80 ± 0.45	1.02	2	0.43 ± 0.20^*∗*^	0.55	4	0.28 ± 0.19^*∗*^	0.36	4	0.28 ± 0.31^*∗*^	0.31	4

Results are expressed as the mean ± SD of at least three experiments. There were the significant different values of skin permeation flux of MMF and MPA, ^*∗∗*^ *p* ≤ 0.05 and ^*∗*^ *p* ≤ 0.05, respectively, when compared with no enhancer of 300 *μ*g/mL MMF solution in each drug.

**Table 3:** The skin permeation flux, ER, and lag time of MMF and MPA at various combination ratios of eucalyptol and N-methyl-2-pyrrolidone.

	No enhancer		Combination ratios of mix enhancers (eucalyptol : N-methyl-2-pyrrolidone) (% w/v)											
	MMF 300 *μ*g/mL		5 : 5			5 : 10			10 : 5			10 : 10		
	Flux (*μ*g/h/cm^2^)	Lag time (h)	Flux (*μ*g/h/cm^2^)	ER	Lag time (h)	Flux (*μ*g/h/cm^2^)	ER	Lag time (h)	Flux (*μ*g/h/cm^2^)	ER	Lag time (h)	Flux (*μ*g/h/cm^2^)	ER	Lag time (h)
MMF	0.23 ± 0.05	12	1.93 ± 0.61^*∗∗*^	8.21	2	1.20 ± 0.22^*∗∗*^	5.13	2	0.20 ± 0.14	0.85	2	1.02 ± 0.78^*∗∗*^	4.37	2
MPA	0.78 ± 0.03	6	1.37 ± 0.18^*∗*^	1.75	2	1.64 ± 0.19^*∗*^	2.10	2	1.07 ± 0.03	1.37	2	0.84 ± 0.04	1.07	2

Results are expressed as the mean ± SD of at least three experiments. There were the significant different values of skin permeation flux of MMF and MPA, ^*∗∗*^ *p* ≤ 0.05 and ^*∗*^ *p* ≤ 0.05, respectively, when compared with no enhancer of 300 *μ*g/mL MMF solution in each drug.
